# The analysis of collective orientation and process feedback in relation to coordination and performance in interdependently working teams

**DOI:** 10.1371/journal.pone.0297565

**Published:** 2024-03-21

**Authors:** Vera Hagemann, Michèle Rieth, Kai N. Klasmeier

**Affiliations:** 1 Faculty of Business Studies and Economics, Business Psychology & Human Resource Management, University of Bremen, Bremen, Germany; 2 Federal Institute for Occupational Safety and Health, Dortmund, Germany; Federal University of Goias: Universidade Federal de Goias, BRAZIL

## Abstract

Effective teamwork is not only essential for teams themselves, but also for organizations and our society. To facilitate team processes and enhance team performance, feedback interventions are a widely used means. However, different types of feedback (i.e., individual vs. team-level feedback, performance vs. process feedback) can have various effects leaving the question of their effectiveness unanswered. This is especially important when team members’ attitudes (namely collective orientation) are considered. Thus, understanding the interplay between types of feedback and team members’ attitudes would reveal new opportunities for fostering reliable teamwork. The methodology of the present study is based on a laboratory approach. Teams (*N* = 142) of two worked together over four scenarios to extinguish forest fires in a microworld. We examined the effects of collective orientation on team coordination and team performance. To understand the interplay between feedback and attitudes we examined the effect of different feedback interventions on team performance and on a change in collective orientation. For analyzing multilevel mediation and changes over time, Bayesian multilevel models were applied. Results show a positive relationship between collective orientation and team performance mediated by coordination. Additionally, team-level process and performance feedback seem to be slightly more beneficial for maintaining performance over time with increasing difficulty of the task compared to individual-level process feedback. Feedback can lead to an increase in collective orientation if these values are low at the beginning. Our research highlights the importance of collective orientation and feedback interventions on team processes and performance for interdependently working teams.

## Introduction

A major proportion of work in associations, voluntary work, organizations, or even science does not take place in individual work but through teamwork. In line with the increasing prevalence of team-based structures in recent decades, research on teams has grown steadily [1–3]. Teams are able to bring together individual competencies and organize work to accomplish tasks and solve problems that could not be solved by individuals [4]. The focus of research nowadays clearly goes beyond the analysis of normal work teams (e.g. production or project teams) under stationary conditions or in virtual environments [5]. Teams are becoming more important, especially for complex problems, in order to successfully solve problems and tasks in critical situations with partly unpredictable events in interdependence. Examples include teams in medicine [6–8], such as emergency care or air rescue, disaster management teams [9], teams in the military [10], space operations [11,12], or fire departments [13,14]. These teams are often called high performance, high responsibility, or extreme teams [5,15–17] and work in so-called high reliability organizations [18]. All these teams have in common that they have to act in close interdependence and under increased demands in a particularly reliable and attentive way in order not to endanger or save their own lives and those of others.

Task interdependence in this context means that the actions of one person have a strong impact on the work process of all team members so that it directly influences the interaction between all members [19]. These interdependent actions and the common goals are a core element of teamwork. Therefore, teamwork is highly characterized by the team’s processes, which are “members’ interdependent acts that convert inputs to outcomes through cognitive, verbal, and behavioral activities directed toward organizing taskwork and achieving collective goals” [20 p357]. Marks et al. [20] proposed a framework in which they distinguished interpersonal, transition, and action processes. The latter processes include, for example, the coordination and monitoring of resources. Coordination means “orchestrating the sequence and timing of interdependent actions” [20 p363] referring to a team process in which the team members contribute to the collective’s goals without suffering from process losses [21]. Since the successful accomplishment of complex tasks by these high responsibility teams is particularly important for the organizations as well as our society due to the serious consequences, they should be supported in their collaboration as best as possible. One possibility to support collaboration and enhance performance in teams during work and outside training settings is to provide feedback to the teams [22–24]. Whereas research has proven the positive impact of performance feedback on team efficacy and performance [22,25,26], research on the consequences of process feedback on team collaboration and performance is still scarce and existing results are inconsistent [22]. None of the previous feedback studies have addressed interdependently working teams in complex tasks that are of particular relevance to our society. Thus, it is not clear whether at least the positive results from previous studies are applicable to them. Furthermore, none of the studies have analyzed team performance in a dynamic manner, “acknowledging that performance is not a static concept” [27 p795]. As the effective work of these teams is particularly important, we aim to explore these development opportunities through feedback in this paper. Firstly, we propose that performance feedback significantly enhances team performance and would like to replicate the results from the normal team literature [cf. 28] using experimental data for a rigorous test of theory [cf. 29]. Secondly, we are interested in investigating the effects of process feedback on team performance and shedding more light on the so far inconsistent findings. More specifically, we distinguish between individual-level process feedback and team-level process feedback in order to be able to analyze possible different effects. We argue that especially in interdependent teamwork contexts, where collaboration is particularly important, individual-level process feedback tends to be a hindrance, and team-level process feedback tends to be beneficial for effective teamwork.

Besides feedback, it is further important to consider input factors, such as the attitudes of team members, as these can also promote collaboration and increase team performance. Attitudes do not only predict team performance, they also have the advantage that they can be influenced by teamwork related prior experience or by interventions [30,31]. Therefore, we would like to look at the complex interplay between different feedback interventions and team members’ attitudes. One attitude that is of significant relevance in teamwork, encouraging team processes and enhancing team performance, is collective orientation (CO) [31,32]. CO is an individual attitude and is defined “as the propensity to work in a collective manner in team settings” [31 p317]. Strongly collectively oriented individuals enjoy team membership and work goal-oriented in a team, contribute effectively to the joint work, and consider what other team members are saying. Since the significance of CO for successful teamwork in interdependent teams has been empirically proven [31,32], it seems reasonable to select people, for example, for high responsibility teams, who have a high level of CO. One problem in organizational selection processes is that the expression of certain attitudes is rarely defined as criteria or validly assessed. Another difficulty is that there are often not enough applicants for a position so it is unusual to find a person who meets all the criteria. I.e., even if CO has been defined as an important characteristic, it may be that it is ultimately dropped in the selection process because other criteria are considered even more important. For these reasons, it would be very useful to find ways of enhancing CO when it is low in the sense of personnel development activities [cf. 33]. Therefore, thirdly, we aim to demonstrate the high relevance of CO for team coordination and performance in interdependent teamwork and replicate effects from previous studies. Furthermore, we would like to show how CO can be changed positively, as teamwork relevant attitudes can be influenced by interventions [30,31]. Thus, fourthly, we aim to investigate feedback as a situational factor to positively change the attitude CO when it is primarily rather low. This would be a sustainable way to positively change the attitude of team members in practice during work. Therefore, we will investigate whether certain types of feedback can impact team-regulatory processes and positively change CO [see 26]. We argue that especially team-level process feedback can make the mutual information needs of the team members recognizable [23,34,35] and thus have a positive effect on CO.

In the following, we start by describing the different types of feedback and subsequently elaborate on the effects of these types of feedback on team performance. Next, we address CO and its effect on coordination and team performance and explain the possibilities of changing CO by means of certain types of feedback. We then test our hypotheses in a sample of 142 teams with Bayesian multilevel models.

### Types of feedback

Feedback takes a significant role in education, science, work, or even in sports as it powerfully impacts development and performance [36–39]. Feedback is the transmission of information to an individual or team by an external actor regarding actions, results, or processes demonstrated during task accomplishment [40]. Feedback typically serves the purpose of modifying behavior and enhancing performance [22]. It is essential to distinguish feedback from debriefing, which involves a “post-experience analytic process” with discussions [41, p. 166], and team reflexivity, which addresses “the extent to which team members collectively reflect upon their teams’ objectives, strategies, and processes” [42, p. 88].

Different aspects of feedback can be distinguished in the literature. One aspect is the level of the provided feedback; thus, it can be given on a *team* or *individual level* [see 23,26,34,43]. Team-level feedback is directed at the entire team, i.e. the focus is on the actions and performance of a team as a whole (e.g., "In the simulation, you did a very good job of looking at your positions and coordinating with each other."); individual-level feedback is directed specifically at individuals, i.e. the focus is on the actions and performance of individuals within a team (e.g., "You took very good care of the water supply for all the units."). A second aspect is the type of feedback, which means addressing either *performance* or *process feedback*. Performance feedback enables individuals to compare their performance against pre-set goals and to reflect on whether, or not, they need to adjust their behavior or goals [26,36,44]. This type of feedback is similar to a status report (e.g., "You discovered all of the three forest fires and were able to extinguish two."). When individuals receive feedback that highlights that their performance is not in line with predetermined goals, they may seek to reduce this discrepancy between behavior and goals through more effort, application of different strategies, or a modification of goals [45]. Summarized, goal-oriented performance should be encouraged and performance-reducing aspects corrected. However, when performance feedback is given, individuals do not receive any information about *how* they might adjust their behavior or strategies in the future. Thus, performance feedback can motivate but is less helpful in terms of the adaptation of concrete actions [22,40]. Process feedback, on the other hand, includes information about the procedures of how a task was effectively accomplished by an individual or in a team (e.g., "It is very good that you have taken into account the wind direction when setting up the firewalls."). Thus, this type of feedback can improve performance and help individuals and teams achieve their goals more effectively by identifying possible ways in which they can adjust their behavior in the future [22,34]. This type of feedback can be further divided into task-related and interpersonal feedback. Task-related process feedback refers specifically to information about demonstrated behaviors and strategies related to the task (e.g., " You did a very good job of keeping the goals of the mission in mind and adjusting your actions accordingly."). Interpersonal process feedback is more descriptive of the social circumstances under which the team pursued joint task accomplishment (e.g., "You listened to each other very well and answered each other’s questions quickly.").

### Feedback and team performance

In team research, the combinations of the different aspects of feedback are analyzed with regard to their influence on self- and team-regulatory processes and team performance [22–24,46]. Although inconsistent findings resulted from various studies, it has been repeatedly proven that performance feedback, also differentiated by team and individual level, has a positive influence on team performance [23,26,43,47]. Individual- and team-level performance feedback enhances individual and collective efficacy, goal tracking on both levels, and motivation, and thus influences intention formation and team performance positively [24,25,46,48]. As individual-level performance feedback increases individual performance, it is especially successful for teamwork when individuals take on specific tasks and the team’s task performance depends on it [22,26,48]. Team-level performance feedback promotes team reflection processes as well as attention to team goals [42,49] and is especially successful for team performance when the team task is interdependent [22,48]. However, if a team task includes interdependent actions and individual task components simultaneously and if individual goals do not conflict with those of the team task, a combination of individual- and team-level performance feedback is also effective in increasing team performance [22,26]. Summing up, team- and individual-level performance feedback is well researched in the teamwork context. Based on these empirical findings, there may be no necessity to distinguish between individual- and team-level feedback. Thus, the present study examines the relation between individual- and team-level performance feedback with team performance in a single condition. Moreover, we aim to re-investigate this relationship with new data and in a new laboratory study and thus make an important contribution to feedback and team research in terms of replicability [50,51]. Since the teams in the present study work on a team task a total of four times in a row and receive feedback each time, we operationalize the classical input-mediator-output-model in team research [52] with multiple teamwork episodes and rounds and analyze the effects of feedback cycles. As teamwork scenarios become more complex and the demands on teams increase, performance improvement means that team performance does not decline over time. Thus:

**H1:** Individual- and team-level performance feedback should positively relate to team performance development over time. Team performance should be at least stable or increase over time (i.e., across scenarios that become more complex).

By contrast, process feedback has been much less frequently analyzed, and studies have come to inconsistent findings with respect to self- and team-regulatory processes and team performance [22]. Individual-level process feedback has not been shown to be significantly correlated to team performance. It is more likely that individual-level process feedback can affect self-regulatory processes such as motivation, intention, and attention to one’s own goals, and through self-referral strategies and effort, it promotes primarily individual performance [22,26]. The relationship between team-level process feedback and team performance seems to be more positive, but the results are still limited. Team-level process feedback directs attention processes away from the individual to teamwork processes [23] and facilitates the focusing of team actions and learning [22,34], thus positively influencing team performance via self- and team-regulatory processes such as motivation, satisfaction, and cohesion. It also seems that the association of task-related team-level process feedback on team performance is stronger than process feedback focusing on the interpersonal aspect [22]. If the interpersonal aspect is considered in team-level process feedback, this can lead to a stronger perception of the (information) needs and (work) contributions of the other team members [35] and thus to more effective coordination behavior within the team. Even though a few studies have shown a positive relationship between team-level process feedback and team performance, no direct relation between task-related process feedback and team performance was found in a study by Prussia and Kinicki [46]. Also, Kernaghan and Cooke [53] could find this positive relationship only in a subgroup of their sample. Only high ability teams showed a direct correlation between task-related process feedback and team performance. One possible explanation of those results could be the teamwork context, especially interdependence in the team task. In this sense, it is often discussed that team-level feedback can be most effective in terms of increasing team performance when teams work interdependently, i.e. team goals are in focus and individual performances cannot simply be added up [22,48]. Without interdependence of team members, an increase in individual performance would be sufficient. In the case of interdependent teamwork, on the other hand, attention processes must be placed on the coordination of team actions so that team goals can be achieved.

Summarized, the differentiated relationships of individual and team-level process feedback on team performance have not yet been validly researched, especially for interdependent teams such as high responsibility teams, that may need feedback to improve their collaboration and avoid critical situations [37,54,55]. In order to illuminate this research gap and to gain new insights regarding individual and team-level process feedback and team performance, the present study explicitly distinguishes these two conditions and analyzes them separately. The few studies conducted to date on individual-level process feedback and team performance have been unable to establish any relationship [22,26]. Since this form of feedback may not be able to enhance team performance, we propose that it could be a hindrance, especially in interdependent teamwork requiring effective communication and coordination between team members [22,48,56,57]. On this basis, we propose that

**H2:** Individual-level process feedback should negatively relate to team performance development over time. Team performance should decrease over time (i.e., across scenarios that become more complex).

Furthermore, “team-level process feedback has been examined scarcely so that any decisive conclusion cannot be given as yet” [22 p136]. The scarce state of research is also emphasized by the limited number of studies with inconsistent findings in the context of process feedback and team performance. Some studies have demonstrated positive effects [22,34], while others have not [46,53]. Studies with positive relationships demonstrate that team-level process feedback is able to direct attention processes to team-regulatory processes [23] and to facilitate the focusing of team actions and learning [22,34]. Even if task-related team-level process feedback would have a stronger relation with team performance than team-level process feedback with a focus on the interpersonal aspect [22], it is important to include this interpersonal aspect in this type of feedback. Especially for interdependent working teams, the consideration of the interpersonal aspect in the feedback is of high relevance to improve their coordination behavior [35]. Given the interdependence present in our team task and the assumption that team-level process feedback can promote team-regulatory processes and thus positively relates to team performance, we propose that

**H3:** Team-level process feedback should positively relate to team performance development over time. Team performance should be at least stable or increase over time (i.e., across scenarios that become more complex).

### Collective orientation, team performance and feedback

CO is an individual attitude that is relevant for teamwork [58], as it improves team performance by coordinating, evaluating, and considering the task contributions of other team members in the accomplishment of a team task [59]. CO is a team composition variable. Team composition has received considerable attention in team research because the configuration of team members’ characteristics has a strong influence on team processes and outcomes [59,60]. The evidence of construct and criterion validity of CO was demonstrated in previous studies [31,60]. The impact of a positively shaped CO on individual behavior in a team context can also be explained on the basis of the collective effort model (CEM) [61]. Based on the CEM, team members with high CO values are more likely to put effort into a team task, they seem more likely to recognize that their individual efforts matter in achieving valuable outcomes and are therefore more successful. To better understand why CO is related to team outcomes, we consider team coordination as an explaining mechanism. A few empirical studies have shown that, on the one hand, CO enhances coordination among team members [30] and, on the other hand, promotes team performance [31,62] through information sharing, strategizing, and goal setting [59]. Moreover, especially in complex, interdependent teamwork contexts, the positive influence of CO on team performance seems to be unfolding [31,62]. Interdependence increases the degree to which team members are co-dependent on each other and requires them to coordinate more effectively with each other, increasing the coordinative complexity [57]. Thus, as people with high CO levels are more motivated to invest their efforts in intra-team interaction, it is reasonable that coordination behavior will be enhanced in interdependent tasks and team performance will increase. Two studies [32,56] exist that have been able to empirically demonstrate this relationship between CO and team performance mediated by coordination. The present study also analyses coordination as a team process variable influenced by CO, as it is our concern to re-analyze this relationship with new data and under altering conditions and thus to make an important contribution to CO and team research in terms of replicability [50,51]. Thus, we propose that

**H4:** Coordination behavior should mediate the relationship between collective orientation and team performance.

Empirical findings indicate that CO is of high importance for effective coordination in the team and thus also for the team’s performance. In many organizations, teamwork is a crucial aspect, and individuals who contribute significantly to it are highly sought after. Therefore, it is of utmost importance for organizations to have team members with a positive CO. However, due to the lack of skilled workers in many industries, individuals cannot be selected for all relevant skills, even for teamwork positions. Therefore, interventions that can promote teamwork-relevant attitudes during work, for example during debriefings or after actions reviews, are important. One possibility in this context can be interventions based on feedback given by supervisors or team leaders. Team-level performance feedback is thought to promote reflection processes as well as attention to team goals [42] and, hypothesized theoretically, can lead to CO [43]. Most importantly, it is known that team-level process feedback directs attention processes away from the individual towards teamwork processes [23] and encourages to focus on team actions and learning [22,34]. If the interpersonal aspect is included in the team-level process feedback, this can lead to a stronger perception of the (information) needs and (work) contributions of the other team members and thus to more CO [35] and thus to more effective coordination in the team. To this point, however, there have been no studies that have empirically examined the theoretical relationship between feedback interventions and a change in CO, although it has already been discussed that team-level process feedback shifts attention processes toward team action and learning [23,34,35] and thus may have the potential to change CO positively. Since research related to team-level process feedback has been very limited [cf. 22], there is a need for research related to team-level process feedback and whether this may lead to an improvement in CO, especially when it is low. On this basis, we propose that

**H5:** Collective orientation should increase from pre to post within the condition of team-level process feedback.

## Method

### Sample

The sample included 284 undergraduate and graduate students from two Universities studying political or social science, business studies, or psychology. The mean age was *M* = 22.79 (*SD* = 4.47; one missing value) and the age ranged from 18 to 49 years. 206 participants were female (73%), 77 were male (27%), and one participant was diverse. Two people formed a team each. Participants either received four hourly credits as a trial subject or received 20 Euros compensation. All people were informed about the purpose of the study and told that they could discontinue participation at any time. Informed consent was obtained from each participant beforehand.

### Team task

The team task was a simulation-based task and is called C^3^Fire [see 63]. C^3^Fire is a command, control, and communication simulation environment in which participants work interdependently [64]. It is a microworld [cf. 65] and the task environment is complex as people strive for (contradictory) goals, processes are coupled, situations change dynamically and are often opaque. Based on these characteristics, C^3^Fire is comparable to tasks people typically come upon in real-life settings [65]. Microworlds such as C^3^Fire are able to bridge the gap between laboratory studies, which can have deficits in terms of ecological validity, and field studies, which have been criticized for their lack of control [see 65]. In C^3^Fire, the team members have to coordinate their actions in order to fight forest fires and they are in charge of protecting lives and houses. The teams consisted of two firefighting units, one mobile water tank unit (accountable for re-filling the firefighting units’ water tanks that they carry with them) and one fire-break unit (a field defended with a fire-break cannot be ignited; the fire spreads around its ends). Each team member was responsible for the same two units in all scenarios, either firefighting and water tank unit or firefighting and fire-break unit. For an illustration of the microworld, see S1 Fig. In total, four different scenarios were developed in the microworld, with increasing complexity. Due to the characteristics of complex systems [65] and the change of some parameters in the C^3^Fire simulation scenarios [63], possible learning effects can be prevented. For example, the fire fighter’s field of vision changes and becomes smaller from the second scenario. From the third scenario on, additionally, a second fire is added, which increases the coordination effort in the team. Finally, a stationary water tank, to replenish the water supply of the emergency forces, is moved to the edge of the playing field, so that the teams need to coordinate more closely with the mobile water tank unit. This led to a significant increase in coordination requirements in the team from scenario 3 onwards. Furthermore, due to the increase in demands on the teams from scenario to scenario, the effectiveness in the team also had to increase in order to show a constant team performance. Communication between the teammates happened by means of a chat system.

### Intervention

The feedback manipulation was the treatment in the present study. In total, three feedback conditions existed. The distinction of feedback conditions was based firstly on the type of feedback (performance or process feedback) and secondly on the level of feedback (team or individual) [cf. 22]. As explained above, performance feedback is already well researched, a condition has been created for this type called team and individual-level performance feedback (PerfTI). The effects previously studied of process feedback are more diverse than those of performance feedback [22,39,66], resulting in two more conditions. One condition is called process feedback team level (ProcT) and the other is called process feedback individual level (ProcI). Feedback was given based on three feedback guidelines so that the feedback is standardized and no information is omitted. The feedback guidelines consist of categories tailored to the task in the microworld. All categories include specific statements that can be marked by the observer if the behavior was shown during task completion. The feedback guideline for PerfTI focused on the performance of both teammates, on an individual and a team level. Only results, i.e. final states of an action, are mentioned. Each category of the guideline includes specific statements with two degrees of fulfillment, one of which could always be checked off and later addressed to the team in the feedback. An example is checking off the statement "You have few burned fields, that was good." or "You have many burned fields, that was not so good yet.". The feedback guideline for ProcT focused on the interpersonal and task-specific behavior shown during task fulfillment and was given on a team level to both teammates. The procedure for this feedback guideline was the same as for the first guideline. An example is checking off the statement "You have shared well about the conditions and availabilities of your units, keep it up." or "You have not shared so well about the conditions and availabilities of your units, try to do better.". The feedback guideline for ProcI focused on the interpersonal and task-specific behavior shown during teamwork of each single team member. It was given on an individual level first to one team member and then to the other one. Regarding this feedback guideline, some statements were only relevant for one player and some for the other player, as they had different subtasks to complete. An example is checking off the statement "You have strategically placed your fire walls well, keep it up." or "You have not strategically placed your fire walls well yet, pay better attention to the spread of fire.". In addition, there were statements that related to common tasks and were relevant to both. But in this case, the behavior shown by the team members can be different, so that, for example, one player receives the positive statement and the other receives the negative one. An example of this is checking off the statement "You asked your partner questions, that was good." or "You asked your partner few questions, try to do better.”. For this feedback condition two guidelines existed, one for each player. For an overview of the three feedback conditions with example statements, please see S1 Table.

### Instrument

We used validated instruments from the literature, which were already tested for their suitability for the C^3^Fire simulation in previous studies [32,56].

*Collective Orientation* was assessed with 16 items [60]. All items were rated on a 5-point Likert scale (1 = *strongly disagree* to 5 = *strongly agree*). An example item is “*I find working on team projects to be very satisfying*”. Cronbach’s alpha for this scale was .84 (pre, T0) and .87 (post, T1).

#### Control variables

*Knowledge about the microworld* was measured with one cloze (three words required, 3 points maximum) and three multiple-choice questions (9 points maximum), developed by the first author. Points ranged between 0 (no correct answer) and 12 (all answers correct). One example multiple-choice question is “Which fields do not ignite? Possible answers: Fields closed out; … burned out; … with a house; … with a gas tank; … with a fire-break; … without trees and bushes”. Since three answers are correct, a maximum of three points can be earned for this question. A knowledge of the microworld and how it works is required to complete the team task. If the participants were to show a lack of knowledge, poor performance could be directly attributable to this and not to the treatment.

*Gaming behavior* was measured with one item “On average, how many hours a week do you spend playing computer games?”. Values ranged between 0 and 100 hours (only one person) with a mean value of 3.19 (*SD* = 6.84). Since gaming behavior could have an influence in such computer-based microworlds on the performance, we also controlled for this.

#### Coordination

Coordination behavior within the teams was assessed as the time the firefighting units spent without water in the field in relation to the total scenario time. This is an appropriate indicator of coordination because it represents the effective exchange of relevant information and resources (sequence and timing) among team members on a reciprocal basis, which is an essential characteristic of effective coordination [67]. This measure represents coordination performance regarding the water replenishment process in the microworld and presents the effectiveness of resource-based coordination [68] and belongs to action processes [20]. The water replenishment process requires coordinated activities between the one water tank unit and the two firefighting units. One team member was in charge of the mobile water tank unit and thus was responsible for the timely refilling of the water tanks of his own fire unit and those of the other team member. To prevent running out of firefighting water, team members had to share information, such as the current and future location of their firefighting units and mobile water tank unit, or even their water levels. The underlying assumption is that a more effective coordination process will result in fewer delays in completing the water replenishment process. Coordination was calculated by the following formula. Values ranged between 0 and 1, with lower values indicating better coordination in the team [see 69].

*Coordination* = time spent without water / total time spent in scenario

#### Team performance

Performance within teams was calculated from a formula that contained the teams’ objectives (limiting the number of cells burned out and protecting as many buildings as possible). The formula includes the number of buildings, fields, and bushes/trees protected relative to the number of buildings, fields, and bushes/trees that would burn in a worst-case scenario. This formula considers that teams that take more time to fight fires also have more burning cells and show less successful performance than teams that are fast at fighting fires. The buildings, bushes/trees, and fields were also weighted according to their different importance in the formula, reflecting the teams’ goals. The primary goal was to protect the buildings. Bushes/trees (medium importance) burned faster than fields (least importance) and favored the spread of fire. Values for team performance ranged between 0 and 7.99. Higher values indicate a better overall performance. Team performance was calculated as follows:

TeamPerformance=((a/maxa)*5)+((b/maxb)*2)+((c/maxc)*1)

a means the number of protected houses (not touched by the fire), b means the number of protected bushes/trees, c the number of protected fields, max a means the number of affected houses in the worst case (houses burned out, extinguished or still on fire), max b the number of affected bushes/trees in the worst case and max c the number of affected fields in the worst case. The numbers 1 to 5 represent weightings, so that houses, for example, are weighted with 5, since they have the highest priority in the task.

### Design and procedure

This study was designed as a laboratory experiment. We used a 2 x 3 factorial design in which the belonging to a group (high or low values in CO) was the between-subjects factor and feedback giving (performance feedback team and individual level, process feedback team level, process feedback individual level) was a repeated-measures factor (four times). The dependent measures were the coordination behavior (assessed as time spend without water in the scenario) and performance (reaching the objectives, assessed with a formula).

**Fig 1:**
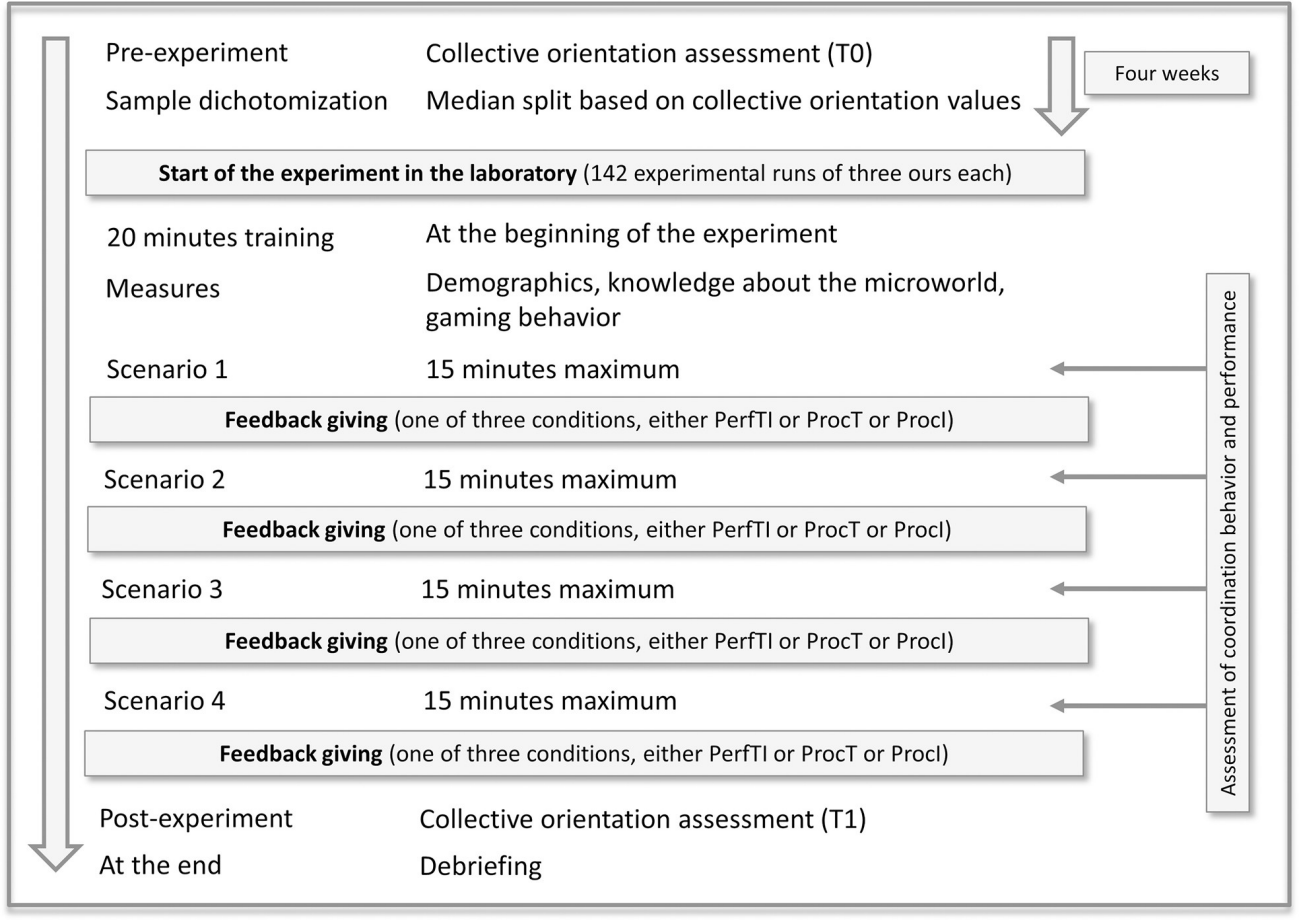
Overview about the experimental procedure.

Prior to the start of the experiment, all participants completed the CO instrument online (pre-assessment). The sample was then dichotomized into two groups with respect to the variable CO using median split (Md = 3.13; range: 1.81–4.94; scale range: 1–5). Two individuals with either low or high CO levels were matched as teammates. Thus, one group of teams (*n* = 74) was characterized by low CO levels and one group (*n* = 68 teams) by high CO levels. Participants were invited to the experimental study three to four weeks after completing the CO instrument. The experimental supervisor scheduled individual appointments with all 142 teams in the laboratory. Each experimental run involved one team, lasted about three hours, and began with an introduction to the experimental procedure and the teams’ task. Participants were first introduced to the simulation, received 20 minutes of training, and completed two practice tests during the training. After the training, participants answered questionnaires measuring demographics, knowledge about the microworld and gaming behavior. At this time, all control variables were collected. Following this, the first simulation scenario started and each team had a maximum of 15 minutes to work on the task. After finishing the scenario, participants received structured feedback from the experimenter, based on one of the three feedback conditions. The experimenter read the feedback statements marked on the feedback guide that matched the participant’s behavior. In the team- and individual-level performance feedback and team-level process feedback conditions, the experimenter did this for the two teammates. The experimenter did this first with one team member, and then with the other team member, when the focus was on individual behavior only within the individual-level process feedback condition. Following, participants repeated the received feedback in their own words for enhancing memorization. It was also a check to see if the participants understood the feedback. Subsequently, participants worked on scenarios 2, 3, and 4 (each lasting for a maximum of 15 minutes). After finishing each scenario, teams received their structured feedback depending on the feedback condition [cf. 70]. At the end of the experiment, CO was assessed again. The experiment finished with a debriefing. The procedure is displayed in Fig 1. The study was approved in advance by the University Ethics Committee of the University of Bremen (UB2019-16).

### Statistical analysis strategy

The data of our study had a multilevel structure of repeated measures based on the four different scenarios (i.e., within-team level or Level 1) nested in teams (i.e., between-team level or Level 2). Therefore, we analyzed the data with Mplus 8.5 [71] using Bayesian multilevel modeling with uninformative priors. To yield unconflated parameter estimates, we applied group-mean centering to our Level 1 variable coordination and added the aggregated group mean on Level 2 [cf. 72,73]. As team performance has been a repeated measure, we used random coefficient growth curve modeling [74] to analyze the development of team performance across the four scenarios. Hence, time has been modeled as linear random slope (i.e., 1 = measure of team performance in scenario 1 to 4 = measure of team performance in scenario 4) on the within-team level. Prior to the analysis, time has been grand-mean centered [73]. As feedback has been manipulated on the between-team level, we examined the influence of feedback on the temporal development of team performance as a cross-level interaction effect [75]. Accordingly, we modeled ProcT and ProcI as two separate dummy variables on the between-team level and used PerfTI as reference category for each dummy variable.

To test our proposed mediational framework (Hypothesis 4), we used a subset of our data based on scenarios 3 and 4. Within these two scenarios, the coordination demands on the teams were distinct, and time spent without water on the field was a valid indicator of coordination. We applied a 2-1-1 multilevel model [see 76] with Bayesian estimator to test the indirect effect of collective orientation (Level 2) on performance (Level 1) via coordination (Level 1). As dependent variable, we used difference scores of CO by subtracting CO pre-experiment values (T0) from the CO post-experiment values (T1). For Bayesian model estimation, we used 100,000 MCMC iterations for each model. Following Depaoli and van de Schoot [77], we evaluated Bayesian model fit by relying on PPC, PPP, PSR, and inspection of trace and autocorrelation plots for every parameter (PPC and PPP are not implemented for multilevel models with random slope in Mplus 8.5). As the autocorrelation plots indicated some degree of autocorrelations, we thinned the posterior distribution by using only every 10^th^ iteration to reduce the degree of autocorrelation.

## Results

Descriptive statistics (i.e., means, standard deviations, and intercorrelations for the within and between-team level) are shown in Table 1. Team performance had an ICC1 of .26 and coordination had an ICC1 of .04.

**Table 1:** Descriptive statistics and intercorrelations.

	*M*	*SD*	2.	3.	4.	5.	6.	7.	8.	9.	10.
**1. Time**	2.50	1.12	-	-	-	-	-	-.24**	-	-	-.10*
**2. Gaming Behavior**	3.19	6.84	-	-	-	-	-	-	-	-	-
**3. Knowledge Test**	10.85	0.82	.06	-	-	-	-	-	-	-	-
**4. ProcT Feedback**	0.35	0.48	.03	.05	-	-	-	-	-	-	-
**5. ProcI Feedback**	0.32	0.47	.00	.05	-.50**	-	-	-	-	-	-
**6. CO Condition**	1.52	0.50	.05	.01	-.02	.00	-	-	-	-	-
**7. Coordination**	0.23	0.14	.04	-.09	.05	-.11	.09	-	-	-	-.11**
**8. CO Pre-Experiment**	3.12	0.48	-.08	-.11	.07	-.07	-.82**	-.11	-	-	-
**9. CO Post-Experiment**	3.23	0.47	-.07	-.11	.10	-.07	-.70**	-.12	.85**	-	-
**10. Team Performance**	4.80	2.26	.28**	.04	-.04	.01	-.12	-.16	.00	.01	-

Between-team level correlations are presented below the diagonal, within-team level correlations are presented above the diagonal, ProcT Feedback: 1 = ProcT, 0 = PerfTI; ProcI Feedback: 1 = ProcI, 0 = PerfTI, CO Condition: 1 = high CO levels, 2 = low CO levels, Coordination = Time without water, *N*_*between-team level*_ = 142, *N*_*within-team level*_ = 568

* *p* < .05

** *p* < .01.

We tested the cross-level interaction effect of feedback conditions on the temporal development of team performance across the four scenarios (Hypotheses 1–3) in one Bayesian multilevel model (Table 2). PSR decreased fast and remained under 1.01 after approx. 4,000 MCMC iterations. Additionally, trace plots for all parameters showed a typical pattern of MCMC convergence indicating a satisfying Bayesian model fit.

**Table 2:** Results from multilevel growth curve model.

Model Path		*B*	Lower 95% CI	Upper 95% CI
**Level 2 –Between-Team Level**				
Collective Orientation	→ Coordination	.19	-.15	.53
ProcI	→ Coordination	-.24	-.65	.18
ProcT	→ Coordination	-.01	-.42	.40
Knowledge Test	→ Performance	.03	-.22	.27
Gaming Behavior	→ Performance	.44**	.20	.67
Collective Orientation	→ Performance	-.36	-.85	.14
Coordination	→ Performance	-.25*	-.50	-.00
ProcI	→ Performance	-.15	-.75	.46
ProcT	→ Performance	-.23	-.82	.36
**Crosslevel Interaction**				
Time x ProcI	→ Performance	-.55**	-.95	-.15
Time x ProcT	→ Performance	-.26	-.65	.14
**Level 1 –Within-Team Level**				
Coordination	→ Performance	-.26**	-.44	-.07
Time	→ Performance	.08	-.21	.35

Presented are unstandardized model coefficients, Coordination = Time without water, Reference for dummy variables ProcI and ProcT is PerfTI

* 95%-CI excludes zero

** 99%-CI excludes zero.

**Fig 2:**
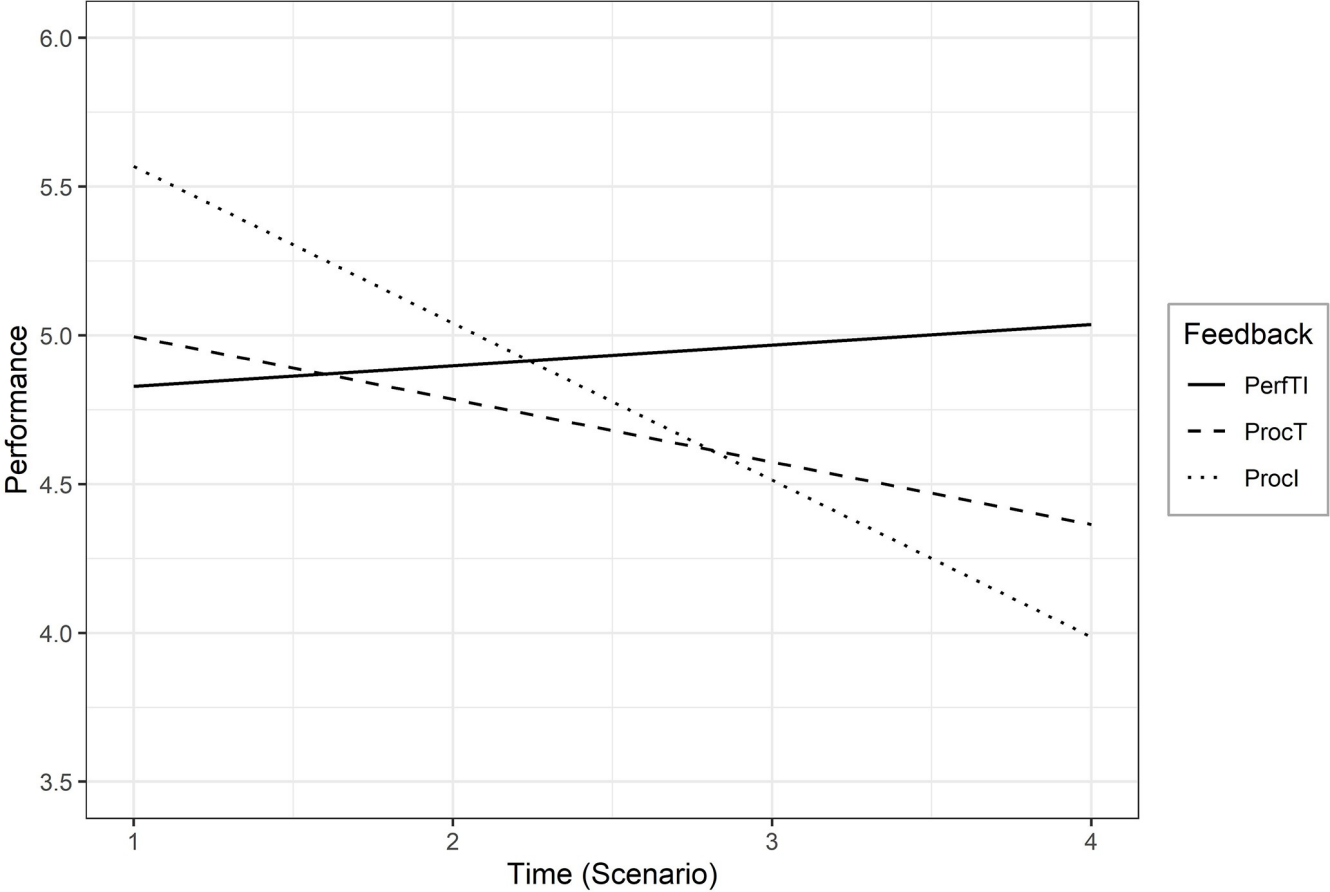
Cross-level interaction effect.

Teams in the PerfTI condition had a constant team performance over the four scenarios with increasing complexity, as the effect of time was likely zero (B = .08, 95% CI = [-.21; .35]). For the teams in the ProcT condition, we did not find a cross-level interaction effect (B = -.26, 95% CI = [-.65; .14]), hence their performance also remained stable over time (B_cond_ = -.19, 95% CI = [-.46; .09]). Accordingly, their development of team performance over the four scenarios was likely similar to the teams of the PerfTI condition. Only the teams in the ProcI condition had a negative development of team performance (B_cond_ = -.49, 95% CI = [-.76; -.19]) indicated by the cross-level interaction effect of ProcI feedback condition on time (B = -.55, 95% CI = [-.95; -.15]). The cross-level interaction effects of the feedback conditions on the temporal development of team performance across the four scenarios are also shown in Fig 2. Summarized, Hypotheses 1, 2, and 3 received full support.

The feedback conditions had no main effects on coordination and team performance, as the posterior distributions of these effects covered zero. Furthermore, coordination was related to team performance on the within-team (B = -.26, 95% CI = [-.44; -.07]) and also on the between-team level (B = -.25, 95% CI = [-.50; -.00]). We tested our mediational framework (Hypothesis 4) in a separate Bayesian multilevel model based on the data of scenarios 3 and 4 (Table 3). Various Bayesian model fit criteria (PPP = .52; PPC 95% CI = [-17.11; 15.91]; PSR < 1.01 after approx. 10,000 MCMC iterations) indicated a good model fit and trace plots exhibited a typical pattern of MCMC convergence.

**Table 3:** Results from multilevel mediation model.

Model Path		*B*	Lower 95% CI	Upper 95% CI
**Level 2 –Between-Team Level**				
Collective Orientation	→ Coordination	.42**	.09	.75
ProcI	→ Coordination	-.09	-.51	.31
ProcT	→ Coordination	-.***05***	-.45	.35
Knowledge Test	→ Performance	-.06	-.36	.24
Gaming Behavior	→ Performance	.50**	.20	.80
Collective Orientation	→ Performance	-.09	-.69	.52
Coordination	→ Performance	-.67**	-.97	-.36
ProcI	→ Performance	-.60	-1.34	.16
ProcT	→ Performance	-.42	-1.17	.29
**Indirect Effect**				
CO → Coordination	→ Performance	-.27**	-.57	-.05
**Level 1 –Within-Team Level**				
Coordination	→ Performance	-.51**	-.81	-.22

Presented are unstandardized model coefficients, Coordination = Time without water, Reference for dummy variables ProcI and ProcT is PerfTI

* 95%-CI excludes zero

** 99%-CI excludes zero.

CO condition was linked to team coordination on the between-team level (B = .42, 95% CI = [.09; .75]) indicating the teams with high CO levels had better coordination behavior. As coordination was related to team performance on the between-team level (B = -.67, 95% CI = [-.97; -.36]), we could also find an indirect relation of CO condition on team performance via coordination (B_ind_ = -.27, 95% CI = [-.57; -.05]). Thus, Hypothesis 4 received support.

We tested Hypothesis 5 in a Bayesian path model and used the difference scores of CO at T0 and T1 as dependent variable. Different Bayesian model fit criteria (PPP = .47; PPC 95% CI = [-10.87; 11.88]; PSR < 1.01 after approx. 3,000 MCMC iterations) indicated a good model fit and MCMC convergence. Descriptive values for CO in all six conditions are shown in Table 4. Feedback condition had no effect on the difference score of CO (B = .22, 95% CI = [-.12; .55] for dummy variable of ProcI vs. PerfTI, and B = .11, 95% CI = [-.22; .44] for dummy variable of ProcT vs. PerfTI). We found an effect of CO condition indicating that teams of the low CO levels condition increased their CO score (B = .18, 95% CI = [.03; .32]). Additionally, we did not find interaction effects between CO condition and feedback condition, as all posterior distributions of the interaction effects included zero as plausible value. In sum, Hypothesis 5 received no support.

**Table 4:** Descriptive statistics of CO values in the six conditions at pre and post assessment.

	PerfTI Feedback		ProcT Feedback		ProcI Feedback	
	T0	T1	T0	T1	T0	T1
**CO high**	3.48 (.30)	3.48 (.46)	3.59 (.34)	3.65 (.41)	3.50 (.27)	3.59 (.45)
**CO low**	2.80 (.31)	2.98 (.38)	2.76 (.45)	2.95 (.51)	2.67 (.31)	2.81 (.39)

CO = Collective orientation; T0 = pre assessment, T1 = post assessment; PerfTI = Performance Team and Individual level; ProcT = Process Team level; ProcI = Process Individual level; *SD* in brackets.

Following recommendations by Bernerth and Aguinis [78], we reanalyzed our models without control variables. The exclusion of controls did not affect the pattern of results, nor did substantially change the magnitude of the unstandardized parameters (differences < |.05|). However, as gaming behavior was positively related to team performance, we included the controls in our results.

## Discussion

This study can increase our knowledge about the relation between various types of feedback and team performance over time in interdependent work contexts while simultaneously considering the interplay between types of feedback and the teamwork relevant attitude CO. Despite our experimental approach, our results and related implications have to be interpreted with caution, as we did not implement a control group (i.e., without feedback). However, this approach may be justified as previous research using the same four scenarios in the C^3^Fire simulation in a different but similar sample could show that no feedback (i.e., a comparable control group) diminished the team performance development over time [32]. Hence, the results of our research may provide evidence about the positive relationship of individual and team-level performance feedback with team performance [22,23,26,43,47]. In particular, we demonstrated that this feedback condition is positively associated with the development of team performance over time [cf. 27] so that team performance remained constant despite the increasing complexity and the accompanying increased requirements on the team. The inclusion of this temporal dimension in the results is of great importance as it provides a nuanced view of how team behavior evolves over time [cf. 79]. Feedback is rarely given by team leaders or others to a team at a single point in time. Rather, teams receive feedback on their performance on an ongoing basis, at different times, so that they can develop and improve [cf. 55].

This research makes an important contribution to the feedback and team performance literature, as it provides a differentiated and contrasting view of possible consequences of team-based vs. solely individual-based process feedback on team performance. Since the few studies on individual-level process feedback could not show any effect on team performance [22,26,34], we have assumed that this feedback condition may be hindering team performance, especially in interdependent work contexts [22,56,57]. In line with this assumption, we have demonstrated that, especially when looking at team performance over time, individual-level process feedback was negatively related to team performance [cf. 27]. This outcome closely resembles the decrease in team performance observed over time when there is no feedback condition, as indicated in the study by Hagemann et al. [32]. Thus, it appears that individual process feedback has no discernible effect beyond that of a no-feedback condition and may therefore be ineffective for interdependent teams.

Because more research is available on team-level process feedback and team performance, with so far inconsistent findings [22,34,46,53], we examined this form of feedback in a third condition. The results are consistent with our hypothesis that process feedback at the team level appears to be positively related to the development of team performance over time, so that team performance remains constant despite increasing complexity and the associated increased demands on the team. This type of feedback may promote team-regulatory processes [23] and team learning [34,42] which are important factors in interdependently working teams and thus positively influence team performance.

Moreover, we were able to replicate the mediational pathway of coordination on the relationship between CO and team performance from two studies [32,56]. Thus, the study contributes to CO and team research by making a robust test of theory with new data and using an experimental approach. Accordingly, team members with high CO appear to be more motivated to understand what information their team members need and thus to interact with them in a goal-directed manner. Thisincreases coordination behavior which in turn is positively related with team performance. As there is a practical relevance to promote teamwork-relevant attitudes such as CO during work and team-level process feedback could be a promising mechanism to achieve that [cf. 42,43], we invested this change in CO from pre to post within this feedback condition. Contrary to the assumption that only team-level process feedback may positively influence team members’ CO when it is low, all three feedback variations were shown to positively relate to team members’ CO when it was low. As team-level performance feedback is thought to promote reflection processes [42] and, hypothesized theoretically, can lead to CO [43] and that team-level process feedback directs attention processes towards teamwork processes [23], our study can provide new empirical insights on those assumptions. There seems to be great room for maneuver so that probably various kinds of feedback are helpful to increase CO when it is low.

However, feedback does not only refer to feedback given by an external person like a supervisor, as in our study, but to information from multiple sources [55]. Especially in high responsibility teams, providing immediate feedback among team members is a critical aspect of increasing reliability and performance [80]. Therefore, future studies may analyze whether the different types of feedback also have the same effect when given by the team members themselves to each other.

### Implications for practice

Process and performance feedback are central to professions with high interdependence, as they can positively influence CO and team performance. Organizations should therefore be at the forefront of their feedback culture and ensure that it is in place at all levels. For example, it would be useful to provide appropriate feedback training for managers and team leaders so that they, firstly, recognize the importance of feedback and secondly, are able to give the appropriate type of feedback in a correct manner.

More specifically, the results may indicate positive effects of performance feedback at all levels and process feedback at the team level, and that process feedback at the individual level does not appear to have any benefit for team performance (is similar to no-feedback, cf. [32]). This reiterates the tremendous importance of appropriate feedback training sessions in which such specifics can be taught.

Especially in high reliability organizations, where teams mostly work together interdependently and maintaining high performance is a priority [18], such as in medicine, air traffic control, fire departments, and police, it should be considered that pure individual process feedback can have negative consequences for team performance. In such organizations, regular mission debriefings, in which feedback is a central component [55,81], are important to promote CO and team performance. Consequently, it would be important to think about designing such debriefings to incorporate feedback, especially performance feedback and process feedback at the team level. In addition, new team members or trainees who are already working with more experienced team members could receive specific feedback at a team level and thus be integrated more quickly into the team processes.

Moreover, high CO has a positive relation with coordination and via this with team performance. Therefore, it seems advisable to integrate CO as a dimension in personnel selection and team composition procedures for occupations where (interdependent) teamwork is important. However, as pointed out before, in practice, this is not always feasible. Our results suggest that CO may appear to be positively modifiable by feedback. Therefore, it seems to be possible that even individuals with initially low CO can strengthen their CO over time through appropriate feedback. Therefore, organizations should encourage this potential positive change in CO through an open feedback culture. Beyond feedback, other interventions might increase CO as well and could therefore be another starting point, such as leadership style and group incentives [cf. 32].

### Strengths and limitations

The experimental manipulation and the simulation setting are suitable to achieve a high internal validity of our findings, as various endogeneity threats are ruled out [e.g., 29]. At the same time, the microworld with its high complexity and dynamics resembles real work environments of interdependent teams very well [65] so that a high ecological validity can be achieved. The sample size can be evaluated as another strength, especially in team research, where data collection in teams involves an enormous amount of time and coordination. Furthermore, the study considered the temporal dimension in regard to the measurement of team performance. Only very few studies have so far considered that team performance is not a static concept [27].

Despite these advantages, there are several methodological limitations associated with the current study design. The study did not include a control group. Therefore, it cannot be clearly stated whether a control group without feedback, for example, would perform as poorly as the teams in the individual-level process feedback condition, or whether a control group would perform even worse or perhaps better. However, the present study was able to show that even teams with low levels of CO in the conditions with performance feedback and team-level process feedback were able to maintain their performance across scenarios of increasing complexity, which has not previously been demonstrated for teams with low CO without feedback interventions [32]. Furthermore, the decline in team performance over time in the individual-level process feedback condition in the present study was similar to the decline in team performance over time without a feedback condition from the study by Hagemann et al. [32]. This study [32] can serve as a quasi-control group for the present study as it used the same four scenarios and the sample was similar in terms of study background, age and gender, and it did not include feedback or other interventions.

Furthermore, the laboratory setting and the students sample precludes external validity and generalizability of our findings. However, the meta-analyses by Kilcullen et al. [33] and Wiese et al. [82] showed that findings on CO and on team performance are equally valid across different sample types (students vs. employees). For these reasons, we assume this comparability also for the effects of feedback. However, future studies could take this into account and investigate it specifically. Despite the realistic nature of our simulation study with a microworld, the operational setting and the consequences of decisions and behaviors of the team are quite different for real high responsibility teams in the field. Nevertheless, also high responsibility teams train their skills in simulations and thus prepare for real missions. In addition, team communication in our experiment was limited to text-based communication, which is not necessarily the same as in the real world. In the real world, people would probably communicate by radio, which would allow other aspects of the team, such as stress, to be better conveyed. Last but not least, we had a special type of team with only two team members. This limits the development of team processes, as, for example, no sub-groups can develop as a result of feedback. In sum, a field-experimental design with a longitudinal data collection may be a promising approach to maximize internal and external validity in future research.

### Conclusion

In summary, the study contributed to the team research literature by providing more evidence on how different types of feedback can be related to team performance in interdependent teams over time while considering the teamwork-relevant attitude of CO. We have shown that it can be useful to distinguish between process feedback at an individual level and at a team level, and have thus been able to show that not every type of feedback may always be beneficial. While performance feedback in general and process feedback at the team level were positively related to team performance, process feedback at the individual level seems to actually hinder team performance in interdependent work contexts. In addition, we provide some evidence that CO can be increased through feedback and strengthen the existing literature by replicating the mediational pathway of coordination in the relationship between CO and team performance. Accordingly, especially in high reliability organizations with high responsibility teams, the consideration of appropriate feedback and the promotion of CO should be of great relevance in order to enable high performing teams.

## Supporting information

S1 Fig User interface of C^3^Fire simulation. For example, for player X, who is in charge of a fire-break unit (grey 4) and a firefighting unit (red 1, placed on a brown square). This scenario contains 16 black houses, one blue water tank, two yellow gas tanks, one black tent and a hospital. Red squares are burning, brown squares are extinguished, grey squares are placed with a fire-break. The visibility window of each unit is marked by the black frame. Fire etc. can only be seen in this area.
(DOCX)

S1 Table Overview of the three feedback conditions with example categories and example statements from the feedback guidelines. (DOCX)
