# Supplementary material for: The analysis of collective orientation and process feedback in relation to coordination and performance in interdependently working teams
Source: PLoS One. 2024 Mar 21;19(3):e0297565. doi: 10.1371/journal.pone.0297565 (PMC10956848; doi:10.1371/journal.pone.0297565)
Supplement: S1 Fig — For example, for player X, who is in charge of a fire-break unit (grey 4) and a firefighting unit (red 1, placed on a brown square). This scenario contains 16 black houses, one blue water tank, two yellow gas tanks, one black tent and a hospital. Red squares are burning, brown squares are extinguished, grey squares are placed with a fire-break. The visibility window of each unit is marked by the black frame. Fire etc. can only be seen in this area. (DOCX) [file pone.0297565.s001.docx]

**Supporting Information**

**S1 Fig.** **User interface of C³Fire simulation.**


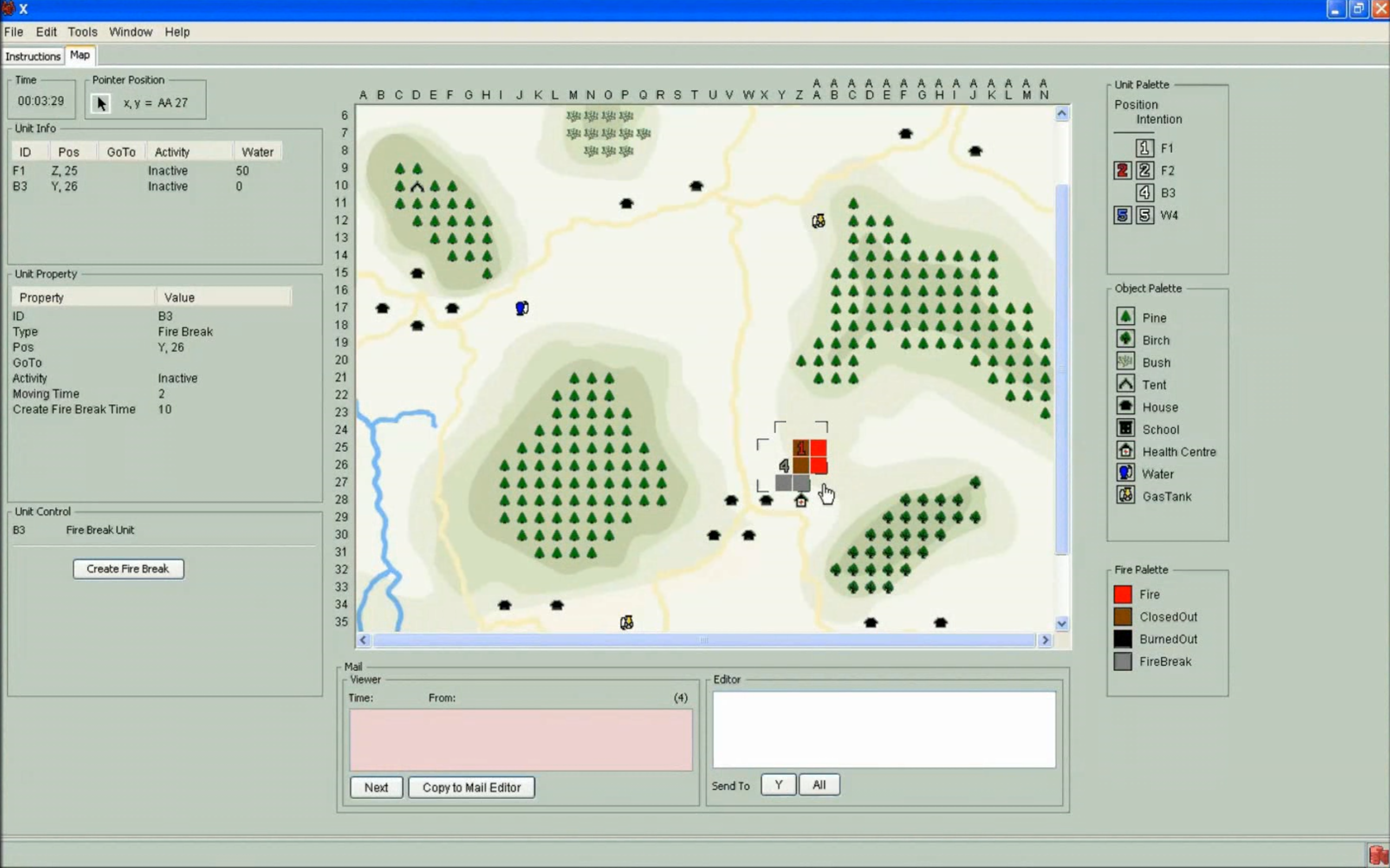


For example, for player X, who is in charge of a fire-break unit (grey 4) and a firefighting unit (red 1, placed on a brown square). This scenario contains 16 black houses, one blue water tank, two yellow gas tanks, one black tent and a hospital. Red squares are burning, brown squares are extinguished, grey squares are placed with a fire-break. The visibility window of each unit is marked by the black frame. Fire etc. can only be seen in this area.
