# Supplementary material for: The analysis of collective orientation and process feedback in relation to coordination and performance in interdependently working teams
Source: PLoS One. 2024 Mar 21;19(3):e0297565. doi: 10.1371/journal.pone.0297565 (PMC10956848; doi:10.1371/journal.pone.0297565)
Supplement: S1 Table — (DOCX) [file pone.0297565.s002.docx]

**Supporting Information**

**S1 Table**. **Overview of the three feedback conditions with example categories and example statements from the feedback guidelines.**

|  | **Process Feedback Team Level** | **Process Feedback Individual Level** | **Performance Feedback Team & Individual Level** |
| --- | --- | --- | --- |
| ***Firebreaks*** |  |  |  |
| **Line-up tactics** | ------- | You (player X) positioned your firewalls strategically well. Keep it up! OR  You (X) positioned your firewalls strategically not very well; pay more attention to the fire expansion. | ------- |
| **Number of walls** | ------- | ------- | You (player X) set many firebreaks; that was good. OR  You (X) set only a few firebreaks; that was not so good. |
| ***Fields*** |  |  |  |
| **extinguished** | ------- | ------- | You extinguished many fields; that was good. OR  You did not extinguish many fields; that was not so good. |
| **burning** | ------- | ------- | At the end of the scenario no fields were burning anymore, that was good. OR  At the end of the scenario some fields were still burning, that was not so good. |
| **burned out houses** | ------- | ------- | 1/2/3/4/5 houses burned down, that was not good. |
| ***Coordination*** |  |  |  |
| **time without water** | In order to fill-up your water tanks you coordinated your actions very well (i.e. you told the other one what you had in mind), so that you spent only a small amount of time without water. Keep it up! OR  In order to fill-up your water tanks you did not coordinate your actions very well (i.e. telling the other one what you have in mind), so that you spent a lot of time without water. Tried to improve your behavior. | You spent only a small amount of time without water and (X) coordinated your actions very well with your teammate / (Y) were on standby. Keep it up! OR  You spent a lot of time without water and (X) did not coordinate your actions very well with your teammate / (Y) were not always on standby. Try to improve your behavior. | You did spend a small amount of time without water; that was good. OR  You did spend a lot of time without water, pay attention to reduce this time. |
| **distribution of units** | You spread your units strategically well, so that you had everything under control. Keep it up! OR  You did not spread your units strategically well, so that you did not had everything under control. Tried to improve your behavior. | You (X, Y) spread your units strategically well, so that you had everything under control. Keep it up! OR  You (X, Y) did not spread your units strategically well, so that you did not had everything under control. Try to improve your behavior. | ------- |
| ***Communication*** |  |  |  |
| **questions asked?** | You asked each other questions; that was good. OR  You asked each other only a few questions, tried to ask more questions. | You (X, Y) asked your partner questions; that was good. OR  You (X, Y) asked your partner only a few questions, try to ask more questions. | ------- |
| **questions responded?** | You responded always the questions of each other; that was good. OR  You did not always respond to the questions of the other one. Tried to improve that. | You (X, Y) responded always the questions of your partner; that was good. OR  You (X, Y) did not always respond to the questions of your partner. Try to improve that. | ------- |
| ***resource-oriented*** |  |  |  |
| **positions** | You asked very well for the positions of the units from the other one. Keep it up. OR  You never/rarely asked for the positions of the units from the other one. Tried to improve that. | You (X, Y) asked your partner very well for the positions of his/her units. Keep it up. OR  You (X, Y) never/rarely asked your partner for the positions of his/her units. Try to improve that. | ------- |
| **water** | You exchanged very well information about your water supply and the filling operation. Keep it up! OR  You never/rarely exchanged information about your water supply and the filling operation. Tried to improve that. | You (X) informed your partner very well about your water supply and the filling operation. Keep it up! OR  You (X) informed your partner never/ rarely about your water supply and were not always on standby. Try to improve that. | ------- |
| ***goal-oriented*** |  |  |  |
| **fire** | You exchanged very well information about the fire spots. Keep it up! OR  You never/rarely exchanged information about the fire spots. Tried to improve that. | You (X, Y) provided your partner very well with information about the fire spots. Keep it up! OR  You (X, Y) never/rarely provided your partner with information about the fire spots. Try to improve that. | ------- |
| **houses** | You exchanged very well information about the protection of houses. Keep it up! OR  You never/rarely exchanged information about the protection of houses. Tried to improve that. | You (X, Y) informed your partner very well about the protection of houses. Keep it up! OR  You (X, Y) never/rarely informed your partner about the protection of houses. Try to improve that. | ------- |
| **Time to finish** | ------- | ------- | You finished the scenario successful and extinguished all fires before running out of time. OR  You were not able to extinguish all fires within 15 minutes. |
